# A Clean Milk Exhibit at the International Congress on Tuberculosis

**Published:** 1908-09

**Authors:** 


### A Clean Milk Exhibit at the International Congress on Tuberculosis.

Preparations are being made by the New York Committee of the International Congress on Tuberculosis to include a Clean Milk Exhibit during the three weeks of the Tuberculosis Exhibition at Washington, from September 21, to October 12, 1908.

The coöperation of a number of public-spirited dairy owners, including Mr. W. W. Law, Jr., Hon. Seth Low, and Mr. V. Everit Macy has been obtained, as also of Commissioner Pearson, Commissioner of Agriculture, Professor Moore, Pathologist, State Veterinary College and Professor W. A. Stocking, Jr., Bacteriologist, Cornell University. It will include not only photographs of dairies, statistical charts and Petri plates of bacteriology of milk and illustrations of tuberculin tests for cattle, but will also have a small working dairy with tuberculin tested cow, skilled attendant, sanitary utensils, shipping cases and all sanitary appliances for the marketing of a clean milk. There will be printed on cardboard a series of aphorisms regarding clean milk. There will be represented graphically the food value of milk as compared with other foods, prepared by the State Department of Agriculture, illustrating the general source of infection of milk with tuberculosis, are being prepared under the supervision of Mr. Law, Jr.

In view of the fact that Mr. Nathan Straus is preparing for the exhibition a duplicate of the pasteurisation plants already erected by him in Heidelberg, Brussels and Berlin, the exhibition at Washington, will afford an excellent opportunity for a comparative study of the two methods of marketing milk: (1) Pasteurised; (2) unpasteurised but clean.

---

Health Commissioner Wende has appointed five doctors as medical examiners for the public schools in Buffalo, whose names are as follows: Dr. Arthur C. Schafer, 753 Michigan street, heading the eligible list; Dr. Franklin W. Burrows, 1236

Michigan street; Dr. Daniel V. McClure, 799 Elk street; Dr. William C. Callanan, 292 Niagara street, and Dr. David E. Wheeler, 391 Delaware avenue.

The appointments were all from the eligible list of the Civil Service Commission and took effect August 16. Dr. Callanan will have charge of the parochial schools.

Miss Rose O'Hara, 146 Rhode Island street, has been appointed a school nurse and will look after pupils discharged from school by the medical examiners and also will see that the physicians' directions are carefully observed. Miss O'Hara's name stood at the head of the civil service eligible list.

---

In the Philippines the army is to conduct a test of the use of underclothing of suitable color to protect the troops against the actinic ray. While the effects of the sun in the Philippines and in the hotter portions of India are said to differ materially, it is believed that it would be wise to have a careful investigation made as to the advantages to be derived from the use of underclothing of a color to protect against this ray. Accordingly, the depot quartermaster at Philadelphia, is now having prepared 5,000 suits of underclothing and as many hat linings of an orange red color. They will be shipped to the Philippines, where careful observation will be made to ascertain to what extent color can be depended on as a protection for troops against actinic rays.

---

According to *The Medical Press and Circular* a red nose is by no means a sign of drunkenness, and is as common among teetotallers as tipplers. Indigestion is responsible almost more than anything else for red noses, while excessive tea drinking is apt to play havoc with the complexion in general and with the nose in particular. Sometimes the congested nose is a sign of some serious disorder of the heart, or it may point to a sluggish circulation. The habit of inhaling cigaret smoke and puffing it through the nostrils may contribute to the external wealth of color.

---

The report that carbolic acid is the most frequently used agent for committing suicide provokes double wonder,—that people choose so unpleasant an instrument and that some means cannot be devised for making it less easy for anybody to procure the poison. At present it seems to be no more difficult to buy that deadly drug than to get so much sugar or salt.

---

The Kaiser, according to a recent cable from Berlin, has subscribed $25,000 to the fund for the Koch Institute for combating tuberculosis. This makes up the $250,000 that it was necessary to procure to obtain the $125,000 promised by Andrew Carnegie.

